# Spontaneous expression of the gene of *KI67* and *P53* in cynomolgus monkeys infected with papillomavirus

**DOI:** 10.14202/vetworld.2022.962-967

**Published:** 2022-04-17

**Authors:** Huda S. Darusman, Sela S. Mariya, Isti K. Sari, Maulida A. Nisa, Kartika Sari, Silmi Mariya, Apon Zaenal Mustopa, Uus Saepuloh

**Affiliations:** 1 Faculty of Veterinary Medicine Bogor Agricultural University, Jl. Agatis, Bogor 16680 Indonesia; 2 Primate Research Center Bogor Agricultural University, Jl Lodaya 2 No 5 Bogor, Indonesia; 3 Primatology Graduate School of IPB University, Jl Lodaya 2 No. 05, Bogor, Indonesia; 4 National Research and Innovation Agency, B.J Habibie Building 15th-24th floor, Jl M.H. Thamrin No.8, Jakarta, Indonesia; 5 Animal Biomedical Sciences Graduate School of IPB University, Jl Agatis, Bogor, Indonesia; 6 Research Center For Biotechnology, National Research and Innovation Agency, Jl Raya Jakarta-Bogor, Indonesia

**Keywords:** animal model, biomarker genes, cervical cancer, nonhuman primate

## Abstract

**Background and Aim:**

Cynomolgus monkeys (*Macaca fascicularis*) develop spontaneous infection of Papillomavirus (PV); thus, potentially beneficial for modeling human PV (HPV) infection study. Contrary to human origin, infection in cynomolgus monkeys does not always show evident clinical symptoms of cervical cancer. The absence of cervical cancer clinical symptoms leads us to investigate the molecular mechanism of the HPV infection in cynomolgus monkeys. This study aimed to investigate the messenger ribonucleic acid (mRNA) expression levels of *KI67* and *P53* genes, majorly known as biomarker oncogenesis of PV infection.

**Materials and Methods:**

The polymerase chain reaction (PCR) technique was used with MY11/MY09 primer to screen PV in cynomolgus monkey, further grouped as positive-PV and negative-PV infection groups. Real-time quantitative PCR was also applied to quantify the mRNA expression levels of *KI67* and *P53* genes in animals.

**Results:**

Increased expression of mRNA level of *KI67* genes was significantly higher in Positive- PV group than negative-PV group. In contrast, the *P53* mRNA expression level increased markedly higher in the negative-PV group than in the positive-PV group.

**Conclusion:**

Our study describes the potential of cynomolgus monkeys as a spontaneous oncogenesis model of PV infection-type. However, we used a limited number of cancer genetic markers. So, further study of other genetic markers is required to prove that cervical cancer could be developed naturally in cynomolgus monkeys.

## Introduction

Cervical cancer is a principal cause of mortality and morbidity globally after breast and colon cancer. The World Health Organization (WHO) reported a global estimate of more than 500,000 women diagnosed with cervical cancer annually [1]. Human papillomavirus (HPV) is the most common pathogen transmitted sexually in women or men. Scientists also have several reports showing that most cervical cancers are caused by HPV infections [2,3].

Strategies for developing preventive and curative methods require a robust clinically relevant model that translationally mimics the pathogenesis of infection. Validation of the model, preferably for *in vivo* studies, should ideally encompass all human phenomenon levels, starting from molecular and genetics up to clinical signs. The infection involves genetic interaction making it necessary to consider the genetic background of the animal models for cervical cancer with HPV infection. Although transgenic mice are the commonly used model for HPV-associated HPV cervical pathogenesis [4,5], a significant limitation exists in the model’s translational ability due to the differences in genetics and cervical pathogenesis between humans and mice.

Nonhuman primates (NHPs) are genetically [6], anatomically, and immunologically [7] similar to humans, representing a valuable model system for viral infection study [8]. In addition, NHPs such as cynomolgus monkeys (*Macaca fascicularis*) and rhesus monkeys (*Macaca mulatta*) also spontaneously develop an infection of HPV and share their infection, further characterized as *M. fascicularis* Papillomavirus (MfPV). Despite the proximity of the species’ physiology, immunology to humans, and spontaneous infection with HPV, HPV infections are not entirely manifested clinically as in patients with HPV [9]. However, to the best of our knowledge, only a few studies describe the gene expression of cynomolgus monkeys with spontaneous Papillomavirus (PV) infections, with particular emphasis on the genes from patients with HPV.

A discovery in the genes of patients with HPV in positively cynomolgus monkeys with spontaneous HPV could add more knowledge of PV’s infection profile in NHP and potentially add the validity of the species as an animal model of PV infection. This study assessed genes of *KI67* and *P53* discovered in patients with HPV and evaluated the expressions in cynomolgus monkeys spontaneously positive to PV. This study predicts the potency of healthy cynomolgus monkeys with positive PV similar to infection HPV associated with cervical cancer in humans.

Therefore, this study aimed to investigate the messenger ribonucleic acid (mRNA) expression level of *KI67* as proliferation and *P53* as a tumor suppressor gene in cynomolgus monkeys to predict the potency of PV infection to cause cervical cancer.

## Materials and Methods

### Ethical approval

The animal husbandry and sampling method procedures were done following the Guide for the Care and Use of Laboratory Animals Guidelines and approved by the Institutional Animal Care and Use Committee of the Primate Research Center (PRC) IPB Addendum Number IPBPRC-19-A012.

### Study period and location

The study was conducted from March to September 2021 at Research Animal Facility Laboratory and Biotechnology Laboratory, Primate Research Center IPB, Bogor, Indonesia.

### Sample collection

Samples were cervical swabs intended to collect deoxyribonucleic acids (DNA) PV material from cynomolgus monkeys. The cervical swabs were collected from 136 adult females of cynomolgus monkeys aged 6 years above and characterized with a dentistry scale of M3/M3. All animals were kept in the animal breeding facility of the Primate Research Center of IPB. In addition, the swabs were collected by cytobrush (OneMed, Indonesia) and dipped into Tris EDTA-NaCL(TEN) buffer media (Sigma Aldrich, Germany) (2 mL Tris HCl 1M pH 7.5; 0.2 mL EDTA 0.5 M; 0.2 mL 5 M NaCl; and 97.6 mL distilled water). Finally, the swabs were stored in a 4°C TEN buffer.

### Screening PV in cynomolgus macaque

#### Extraction of DNA and amplification PV using polymerase chain reaction (PCR) technique

The samples were initially analyzed using the PCR method in dividing the animals into “Positive-PV” and “Negative-PV” groups. The DNA extraction for PV was carried out using the QIAmp DNA Blood Mini Kit (Qiagen, Hilden, Germany), following the company’s instructions. The PCR reaction mix for identified HPV comprised 10 pmoL primers forward and reverses, Gotaq master mix PCR (Promega, USA), nuclease-free water, and DNA as a template. The primer used in this study is shown in Table-1 [9-11]. A total of 5 uL of DNA was amplified in a thermal cycler (VerityApplied Biosystem, USA) with PCR condition 95°C for 4 min, denaturation 95°C for 30 s, annealing 54°C for 30 s, and extension 72°C for 30 s. Stage denaturation until the extension was repeated to 40 cycles with amplification using electrophoresis gel agarose 1.8% later visualized in GelDoc2000. In screening with PCR, 27 cynomolgus monkeys consisting of 18 samples of positive infected PV and 9 samples of negative infected PV were used for *KI67* and *P53* gene expression, which started with the extraction of mRNA, followed by real-time quantitative PCR technique (RT-qPCR).

**Table 1:** Primer used in this study

Primer	Nucleotide Sequences (5’-3;)	Reference
MY11	GCCCAAGGCCACAACAATGG	[9]
MY09	CGACCCAAGGGAAACTGGTC	
*KI67* Forward	GGGTCTGAATCGGCCTCATA	[10]
*KI67* Reverse	GCAAGGGTCACAGTTAAGGC	
*P53* Forward	CCGCAGTCAGATCCTAGC	[11]
*P53* Reverse	AATCATCCATTGCTTGGGACG	

### *KI67* and *P53* gene expression

#### RNA extraction and synthesis cDNA

The total RNA was extracted using Zymo Direct Zol (Zymo Research, USA) following the manufacturer’s protocol. The 5 ng/uL RNA concentration was used as a template for complementary DNA (cDNA) synthesis. This process was carried out by a Sensifast cDNA synthesis kit (Meridian Bioscience, Tennessee, USA). The reverse transcription (RT) mix consists of 4 uL×5 Trans Amp Buffer, 1 uL RT enzyme, 5 uL nuclease-free water, and 10 uL RNA. The mix was incubated in a thermal cycler at 25°C for 10 min, 42°C for 15 min, 85°C for 5 min, and 4°C for 2 min.

### mRNA expression level using RT-qPCR

The differential mRNA expression levels of *KI67* and *P53* genes were measured using the Real-time PCR technique (RT-qPCR). This RT-qPCR (Applied Biosystem, QuanStudio 5) used cDNA as a template for differential mRNA expression analysis using Ssofast Evagreen Master Mix (Bio-rad, USA) and a specific primer for *KI67* and *P53* (Table-1). The RT-qPCR mix comprised 1 uL pair of primer, 10 uL Ssofast evagreen SYBR mix, 6 uL nuclease-free water, and 2 uL cDNA. In addition, the setting condition of PCR was initial denaturation at 95°C for 5 min proceeded by 40 cycles of 94°C for 30 s as denaturation, and 57°C for 30 s as annealing, extension, and data collection. The expression of the b-actin gene was used as a reference gene using the 2^-DDCt^ method for relative quantitative (RQ) analysis. Minitab software (Minitab LLC USA) analyzed the RQ data with statistical descriptive and *t*-test methods.

## Results and Discussion

**Figure-1:**
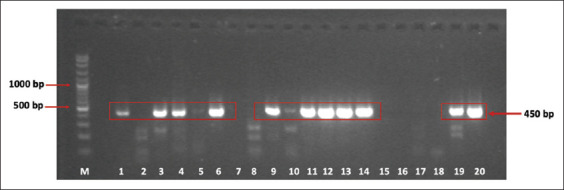
Polymerase chain reaction result screening human papillomavirus (HPV) in cynomolgus macaque. Several samples showed a positive band of HPV (450 bp). The prevalence of HPV in this study is 27.2% (37 of 136 samples positive HPV). Note: (M) Marker 100 bp; (1), (3), (4), (6), (9), (11), (12), (13), and (14) describe band-positive HPV samples; (2), (5), (7), (8), (10), (15), (16), and (17) describe band-negative samples HPV; (18) negative control; (19) DNA-positive control HPV from HeLa Cell.

**Figure-2:**
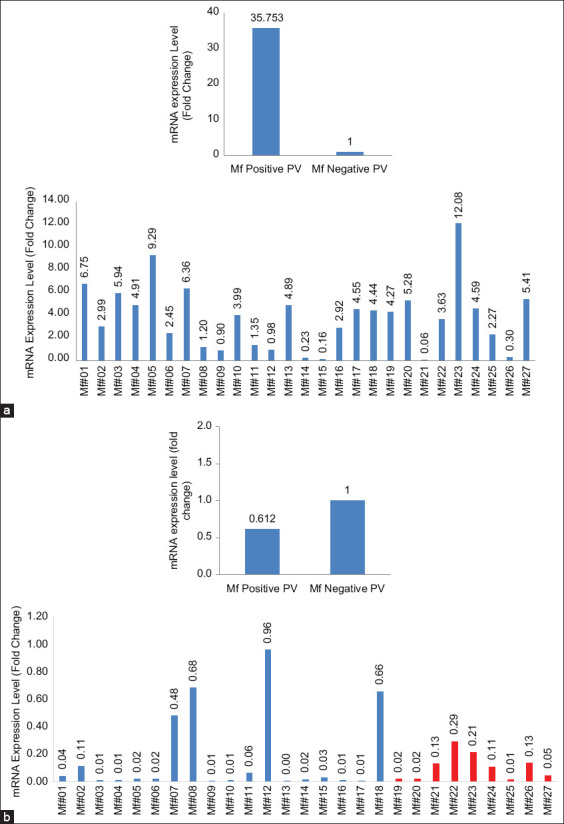
Levels of messenger ribonucleic acid (mRNA) expression for (a) *KI67* and (b) *P53*. Upregulated mRNA expression level in the positive human papillomavirus (HPV) group for *KI67* (p=0.298). The expression *P53* (p=0.395) in the positive HPV group tends to be lower than in the negative group. No significant difference was observed. Values were means, error bars = standard deviation. Blue for the individual with Positive HPV, red for individual with Negative HPV.

The presence of PV in cynomolgus monkeys was identified by PCR technique. We found that 37 of 136 cynomolgus monkeys have been infected with PV (Figure-1), indicating that the prevalence of PV-positive samples was 27.2% in our cynomolgus monkeys captive breeding. Further characterization using nucleotide sequencing showed that these PVs’ subtype was MfPVs (unpublished data). The histogram (Figure-2) shows that the mRNA expression level of the *KI67* gene in the positive-PV group was higher than in the negative-PV group. However, the gene expression of the *P53* gene in the positive-PV group tends to be lower than in the negative-PV samples.

This study evaluated *KI67* and *P53* gene expression to predict cervical cancer in cynomolgus monkeys infected with PV. A total of 37 of 136 animals tested for PV showed positive results (27.2%). Furthermore, low infection prevalence PV was detected, and no symptoms of human PV were seen in cynomolgus monkeys with positive PV. The mRNA expression level of the *KI67* gene as a biomarker of proliferation was higher in positive-PV samples. Interestingly, the mRNA expression level of the *P53* gene as a biomarker of apoptosis in positive PV was lower than in negative-PV cynomolgus monkeys. This study proved that cynomolgus monkeys were naturally infected with PVs and potentially developed cervical cancer as in human PV infection.

The Late 1 (L1) gene is the region that forms the major capsid of the papillomavirus; this protein will be expressed at the end of its formation of virions that occur in the superasal layer of the skin. This area of consensus (consensus region) is usually used for virus identification. The primer pair used in this study is intended to amplify the nt 6582-7033 area and produce a PCR product of 450 base pairs [12]. The primer MY09/011 is extensively used to study the natural history of the papillomavirus and its role in becoming cancer in the genital area. Furthermore, this primer is widely used for various important cervical cancer and HPV studies.

Based on the results of virus identification using PCR techniques in cervical swab samples, the incidence of papillomavirus infection in cynomolgus monkeys in the Pusat Studi Satwa Primata-IPB captive facility was 27.2% (37/136). Wood *et al*. [9] stated that molecular identification of papillomaviruses at the Wake Forest University facilities was 35% in female cynomolgus monkeys. The study also showed HPV infection in female adult cynomolgus monkeys in Indonesia at the middle level between Wake Forest and China facilities. Ki-67 is a nonhistone nuclear protein, and its expression can be observed in cells from the G1 phase of the cell cycle to mitosis. Protein Ki-67 is considered a good indicator of cell proliferation and correlates with tumor growth [13,14]. Cell proliferation is one of the main factors in the biological mechanisms of oncogenesis; therefore, several studies have shown that the activity of cell proliferation is linked with the prognosis of certain cancers such as cervical cancer, breast cancer, carcinoma of the penis, and larynx [15-18]. Ki-67 protein has also been reported to correlate with tumor aggressiveness and has prognostic and predictive value in breast and colon cancer, melanoma, and lymphoma patients [19,20]. Baak *et al*. [21] have evaluated the upregulation of gene encode Ki-67 associated with a severity level of cancer. This research also used female adult cynomolgus macaque, which showed no cervical cancer symptoms. Based on several reported studies, this study has shown that an increased mRNA expression level of the *KI67* gene in cynomolgus monkeys PV has the potency to develop cell proliferation to be a cervical cancer cell.

The mRNA expression level of the *P53* gene in cynomolgus monkeys with HPV was lower than mRNA expression in cynomolgus monkeys without HPV. It can occur since conditions such as DNA damage will increase *P53* protein levels to upregulate apoptosis or terminate the cell cycle. Furthermore, the E6 protein from PV intervenes in this process by binding to *P53* and E6-associated protein ligase, causing ubiquitinylation, and degradation of *P53* [22]. This phenomenon is consistent with the above results; the positive group PV has gene expression of *P53* lower than the negative group. The *P53* protein is a cyclin-dependent kinase (CDK) inhibitor, inhibiting cyclin complexes CDK2 and CDK4 and causing cell cycle termination in the G1 phase. The increased or decreased *P53* expression is considered a prognostic factor in types of neoplasms such as head and neck [23], lung cancer [24], and brain tumors [25]. Khan *et al*. [26] reported downregulation of mRNA expression level of the *P53* gene in humans with cervical cancer in Northeast India, indicating the malfunction of protein *P53*, the tumor suppressor gene. In line with this study, downregulation of the *P53* gene expression in cynomolgus monkeys may be described for future potency malfunction of protein P53 as the tumor suppressor gene, which obstructs the progress of apoptosis. In addition, the decreasing apoptosis progress in an individual with positive PV, with downregulated gene expression of *P53*, has the potency to develop cervical cancer in cynomolgus monkeys.

## Conclusion

This preliminary study proved that cynomolgus monkeys were naturally infected with MfPV subtypes 3 (unpublished data) and potentially developed cervical cancer as in human PV infection. However, we used a limited number of cancer genetic markers. Therefore, further study on other genetic markers is required to prove that cervical cancer could be developed naturally in cynomolgus monkeys. In addition, this study supports the translational potential of cynomolgus monkeys as an animal model for drug and vaccine development against HPV infection.

## Authors’ Contributions

HSD, AZM, US, SM, and SSM: Designed and supervised the study. IKS, US, SM, SSM, and HSD: Sample collection. HSD, SSM, IKS, MAN, and KS: Laboratory work and drafted the manuscript. SSM, KS, AZM, US, SM, and HSD: Revised the manuscript. All authors read and approved the final manuscript.
